# Heterogeneity of IKZF1 genomic alterations and risk of relapse in childhood B-cell precursor acute lymphoblastic leukemia

**DOI:** 10.21203/rs.3.rs-5292018/v1

**Published:** 2024-11-11

**Authors:** Charles Mullighan, Ruth Wangondu, Emily Ashcraft, Ti-Cheng Chang, Kathryn Roberts, Samuel Brady, Yiping Fan, William Evans, Mary Relling, Kristine Crews, Jun Yang, Wenjian Yang, Stanley Pounds, Gang Wu, Meenakshi Devidas, Kelly Maloney, Leonard Mattano, Reuven Schore, Anne Angiolillo, Eric Larsen, Wanda Salzer, Michael Burke, Mignon Loh, Sima Jeha, Ching-Hon Pui, Hiroto Inaba, Cheng Cheng

**Affiliations:** St Jude Children’s Research Hospital; St Jude Children’s Research Hospital; St Jude Children’s Research Hospital; St. Jude Children’s Research Hospital; St. Jude Children’s Research Hospital; University of Utah; Department of Computational Biology, St. Jude Children’s Research Hospital; St. Jude Children’s Research Hospital; St. Jude Children’s Research Hospital; St. Jude Children’s Research Hospital; St. Jude Children’s Research Hospital; St. Jude Children’s Research Hospital; St. Jude Children’s Research Hospital; St Jude Children’s Research Hospital; St. Jude Children’s Research Hospital; University of Colorado School of Medicine; HARP Pharma Consulting; Children’s National Hopital; Servier Pharmaceuticals; Maine Children’s Cancer Program; Walter Reed National Military Medical Center; Medical College of Wisconsin; Seattle Children’s Hospital; St Jude Children’s Research Hospital; St Jude Children’s Research Hospital; St. Jude Children’s Research Hospital; St Jude Children’s Research Hospital

## Abstract

Genomic alterations of *IKZF1* are common and associated with adverse clinical features in B-ALL. The relationship between the type of *IKZF1* alteration, disease subtype and outcome are incompletely understood. Leukemia subtype and genomic alterations were determined using transcriptome and genomic sequencing and SNP microarray in 688 pediatric patients with B-ALL in St. Jude Total Therapy 15 and 16 studies. *IKZF1* alterations were identified in 115 (16.7%) patients, most commonly in *BCR::ABL1* (78%) and *CRLF2*-rearranged, *BCR::ABL1*-like B-ALL (70%). These alterations were associated with 5-year cumulative incidence of relapse (CIR) of 14.8 ± 3.3% compared to 5.0 ± 0.9% for patients without any *IKZF1* alteration (*P* < 0.0001). *IKZF1* deletions of exon 4–7 (*P* = 0.0002), genomic *IKZF1*^plus^ with any *IKZF1* deletion (*P* = 0.006) or with focal *IKZF1* deletion (*P* = 0.0007), and unfavorable genomic subtypes (*P* < 0.005) were independently adversely prognostic factors. Associations of genomic *IKZF1*^plus^ and exon 4–7 deletions with adverse outcomes were confirmed in an independent cohort. Genomic *IKZF1*^plus^ with any *IKZF1* deletion, *IKZF1* deletion of exon 4–7, and unfavorable subtype confer increased risk of relapse. The type of *IKZF1* alteration, together with the subtype, are informative for risk stratification and predict response in patients with B-ALL.

## INTRODUCTION

Five-year survival rates for pediatric patients with acute lymphoblastic leukemia (ALL) exceed 90%, however relapses affect 10–20% of patients and remain a leading cause of death (1–5). Alterations in the *IKZF1* tumor suppressor gene, encoding the lymphoid transcription factor IKAROS, are associated with increased risk for relapse even in the context of risk-directed therapy (6–11).

Somatic *IKZF1* alterations are present in up to 15% of B-ALL cases and are heterogeneous, including broad and intragenic deletions and sequence mutations (7, 9, 12–17). Deletions of exons 4–7 (Δ4–7) are the most common focal *IKZF1* alteration, and result in the expression of the dominant negative IK6 isoform, which lacks the N-terminal DNA binding domains but retains the C-terminal dimerization domains (6, 12, 15). To refine the predictive power of *IKZF1* alterations in B-ALL, the *IKZF1*^plus^ composite genotype has been described (7). It utilizes targeted DNA copy number profiling by multiplex-ligation dependent probe amplification (MLPA) assays to identify *IKZF1* deletions co-occurring with deletions of either *PAX5*, *CDKN2A*, *CDKN2B* or the pseudoautosomal region 1 (PAR1) at Xp22.33/Yp11.31 (as a surrogate for *P2RY8::CRLF2*), which are enriched in high risk leukemia subtypes such as *BCR::ABL1*-like ALL; but excludes cases with deletion of *ERG*, which is common in favorable risk *DUX4*-rearranged (*DUX4*r) ALL (7, 8, 10, 18, 19).

Although *IKZF1* alterations are associated with poor outcome overall in ALL (6, 9, 11, 20–28), and several reports suggest that the *IKZF1*^plus^ genotype identifies a higher risk group of patients than those with *IKZF1* alterations alone (7, 18, 19), the relationship between *IKZF1* alterations and outcomes remains incompletely understood for several reasons. Many studies have been limited to specific subtypes of leukemia or risk groups or have relied solely on the MLPA assay, which does not detect all *CRLF2* (*CRLF2*r) or *DUX4* (*DUX4*r) rearrangements, and may overcall *IKZF1* alterations by including those cases with aneuploidy of chromosome 7 (7). Moreover, most studies have not considered the full molecular landscape of B-ALL defined by recent genomic studies (29, 30). Thus, the full nature of interaction between the type of *IKZF1* alteration, concomitant genetic alterations, and genomic subtype in the context of contemporary risk-adapted therapy is not understood.

Here, using genomic analyses to characterize a wide spectrum of alterations and genetic subtypes of B-ALL, we studied the impact of *IKZF1* alterations on the outcome of pediatric patients enrolled in the St. Jude Total Therapy 15 and 16 studies. Associations between *IKZF1* alterations, subtypes and outcomes were also examined in a cohort of children with NCI standard risk (SR) B-ALL or high-risk (HR) B-ALL, with favorable cytogenetic features, from the Molecular Profiling to Predict Responses to Therapy (MP2PRT) study (31) We identified the combination of *IKZF1* Δ4–7 and unfavorable genomic subtype and genomics-based definition of *IKZF1*^plus^ with focal *IKZF1* deletions as optimal predictors of relapse in children with B-ALL.

## DESIGN AND METHODS

### Patients, risk stratification, and diagnosis

Patients enrolled on Total therapy 15 (Total 15)(2), (NCT00137111 (n = 498) from June 2000 to October 2007) and on Total therapy 16 (Total 16) (32), NCT00549848 (n = 598) from October 2007 to March 2017. Eligibility for this study was assessed for 916 children with B-ALL (Fig. S1) with 688 patients, for whom complete sequencing data were available, selected for study (Table S1). Study protocols were approved by the institutional review boards of St. Jude Children’s Research Hospital and Cook Children’s Hospital. Studies were conducted in accordance with the Declaration of Helsinki. Written consent was obtained from the parents or guardians and assent from the patients, as appropriate.

For both Total studies, low-risk presenting features were age between 1 and 10 years at diagnosis with white blood cell count (WBC) of < 50 × 10^3^/μL, DNA index ≥ 1.16 as a surrogate for high hyperdiploidy, or the presence of *ETV6::RUNX1*, without the following: *TCF3::PBX1*, hypodiploidy (< 44 chromosomes), testicular leukemia, CNS-3 status (≥ 5 leukocytes/μL present in the cerebrospinal fluid with blasts or cranial palsy) at diagnosis, minimal/measurable residual disease (MRD) ≥ 1% on day 19 (Total 15) or day 15 (Total 16) of induction or MRD ≥ 0.01% at end of induction (EOI). Patients with *BCR::ABL1*, any patients with EOI MRD ≥ 1% or persistent MRD during the consolidation phase were classified as having high-risk ALL. In Total 16, infants with *KMT2A* rearrangement (*KMT2A*r) were also considered high-risk. All other patients were considered to have standard-risk ALL. MRD was monitored by flow cytometry.

The MP2PRT study group consisted of 1496 pediatric patients with predominantly SR B-ALL, 1360 patients from AALL0331 (33, 34) and AALL0932 (35, 36) with favorable and neutral cytogenetics) or HR B-ALL, 115 patients from AALL0232 (4, 5) and AALL1131 (37) with favorable cytogenetics (31) (Table S2 and Supplemental methods). All subtypes studied in the Total 15 and 16 studies except for BCR::ABL1, low hypodiploid and CDX/UBTF were represented in 1475 patients selected for the MP2PRT validation study group.

#### Definitions of IKZF1^plus^ and IKZF1 deletions

Genetic and genomic analyses are described in supplementary methods. Three definitions of *IKZF1*^plus^ were used, varying based on the size of *IKZF1* deletion (Δ*IKZF1*) and the comprehensiveness of detection of *CRLF2* and *DUX4* rearrangement were used: 1) “genomic *IKZF1*^plus^ (focal Δ*IKZF1*)” considers focal [up to 20 Mb] *IKZF1* deletions only and includes all *CRLF2* rearrangements (*CRLF2*r*), PAX5* deletions or homozygous *CDKN2A/CDKN2B* deletion, and excludes all cases with *DUX4*r; ; and 3) “MLPA-based *IKZF1*^plus^ (any Δ*IKZF1*)”, with any *IKZF1* deletion (including − 7/del(7p) and focal deletions) and PAR1 deletion, as a surrogate for *CRLF2*r, or deletions of *PAX5* or homozygous *CDKN2A/CDKN2B* deletion, and excludes cases with *ERG* deletion, as a surrogate for *DUX4*r (Table S3). Definitions of genomic subtypes for B-ALL (Table S4) have been previously described (16, 29, 38, 39).

Compared with 228 patients with B-ALL in the Total 15 and 16 cohorts who did not have sufficient genomic data, and were therefore excluded from the final analysis cohort, the studied cohort of 688 patients had a lower frequency of patients identifying as being of white race (79.4% vs 83.3%), a higher frequency of patients of black race (15.4% vs 8.3%; P = 0.009), and a greater proportion of patients with presenting peripheral blood white cell count of ≥ 10,000, including those with WBC ≥ 100,000; 8.7% vs 4.4%; *P* < 0.0001 (Table S5). All other clinical features were comparable between the two groups (Table S5). Differences in the distribution of genomic subtypes were inclusion of all patients with *BCR::ABL1* B-ALL in the studied cohort and a higher percentage of patients with incompletely sequenced or undefined genomic subtypes (B other) in the unstudied group (Table S6, *P* = 0.0004).

### Statistical Analysis for the Total 15 and 16 study groups

Associations between *IKZF1* alterations and other variables were assessed by Chi squared or Fisher’s exact tests as appropriate. Event-free survival (EFS) was defined as the time from diagnosis to the date of last follow-up in complete remission (censored time) or first adverse event. Adverse events included relapse (hematologic, isolated CNS, CNS and ocular relapse, testicular, or any combination), secondary malignancy, lineage switch, or death of any cause. Early death during remission induction and nonresponse to induction therapy were considered as events at time zero. EFS distributions were estimated using the Kaplan-Meier method and compared using the log rank test. The cumulative incidence of any relapse was calculated with the method of Kalbfleisch and Prentice (accounting for competing risk) and was compared between groups using Gray’s test. Cox regression models were used to estimate hazard ratios and assess independent effects of prognostic factors. All multivariable regression models included a time interaction term to account for time-dependent covariate effects. All statistical analyses were performed and illustrated using SAS 9.4 and RStudio version 4.1.2.

## RESULTS

### Frequency of IKZF1 alterations among B-ALL subtypes

In the Total 15 and 16 selected study group, alterations in *IKZF1* were detected in 16.7% of patients (115 out of 688), 13.2% with *IKZF1* deletions (both focal and − 7/del(7p)) (Fig. S2, A), 2.9% with *IKZF1* mutations (Fig. S2, A and C) and 0.6% with a combination of deletions and mutations (Fig. S2, A). *IKZF1* R162P was present in the germline whereas all other mutations were somatic (Fig. S2, C). Among 56 patients with focal Δ*IKZF1*, Δ4–7 deletions were most frequent (32.1%). Other types of focal deletions such as deletion of the entire *IKZF1* locus, and other focal deletions, were each present in less than 15% of patients with focal deletions. *IKZF1* Δ4–7, non-*IKZF1* Δ4–7 focal deletions, and − 7/del(7p) were present in 2.6%, 5.5%, and 5.1% of all 688 studied patients, respectively (Fig. S2, A). Genomic *IKZF1*^plus^ (any Δ*IKZF1*) was detected in 44 (6.4%) patients (Fig. S2, B).

Patients in the Total 15 and 16 selected study group were classified into outcome-based subtype groups. Subtypes with 5-year EFS ≥ 94% were *DUX4*r, *ETV6::RUNX1*, hyperdiploid, and intrachromosomal amplification of chromosome 21 (iAMP21) and were grouped as favorable. Notably, patients with the iAMP21 subtype in the studied cohort had 5-year EFS of 100% without any relapse. *BCR::ABL1*-like without *CRLF2*r, *ETV6::RUNX1*-like, near haploid, *PAX5*alt, and *TCF3::PBX1* subtypes, each associated with 5-year EFS ranging between 80% and 93%, were grouped as intermediate. *CRLF2*-rearranged *BCR::ABL1*-like, *BCR::ABL1*, low hypodiploid, and *KMT2A*r, each associated with 5-year EFS of 50–79% were classified as unfavorable. With these criteria, outcomes for patients with defined subtypes in our cohort were divided into favorable (5-year EFS 95.8 ± 1% and CIR 3.5 ± 0.9%), intermediate (5-year EFS 87.3 ± 3% and CIR 8.7 ± 2.5%), and unfavorable subgroup (5-year EFS 67.7 ± 5.4% and CIR 21.6 ± 4.8%, *P* < 0.0001) (Fig. S3).

**Figure 1:**
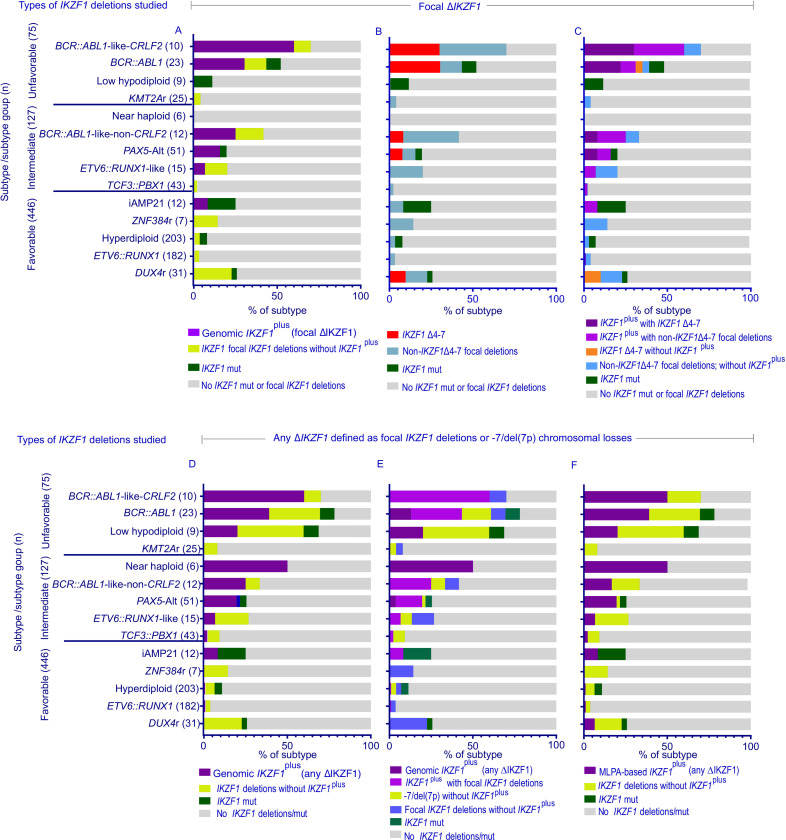
Frequency of *IKZF1* alterations within B-ALL genetic subtypes, in eligible patients with B- ALL in the Total15 and 16 study group (n=688). Color-coded stacked horizontal bar graphs for which segments within each bar represent the proportion of patients (frequency shown on X axis) within respective subtypes (indicated on the Y axis), with color key below each graph, are shown. **A-C**, Focal *IKZF1* deletions and mutations are included. **D-F**, Focal *IKZF1* deletions and −7/del(7p) chromosome losses are included. Genomic *IKZF1*plus includes all *CRLF2*r and *DUX4*r in *IKZF1*plus profile (A, C, and D-F). In MLPA-based definition for *IKZF1*plus, PAR1 deletion serves as surrogate for *P2RY8::CRLF2* fusions and *ERG* deletions are a surrogate of *DUX4*r. B-Other (n = 34) and rare subtypes (*BCL2/MYC*, *CDX/UBTF*, *IKZF1* N159Y, *TCF3::HLF*, and *ZEB2/CEBP*; n = 6) detected in 2 or fewer patients are not included. Mut: missense, nonsense, or frameshift mutations.

**Figure 2:**
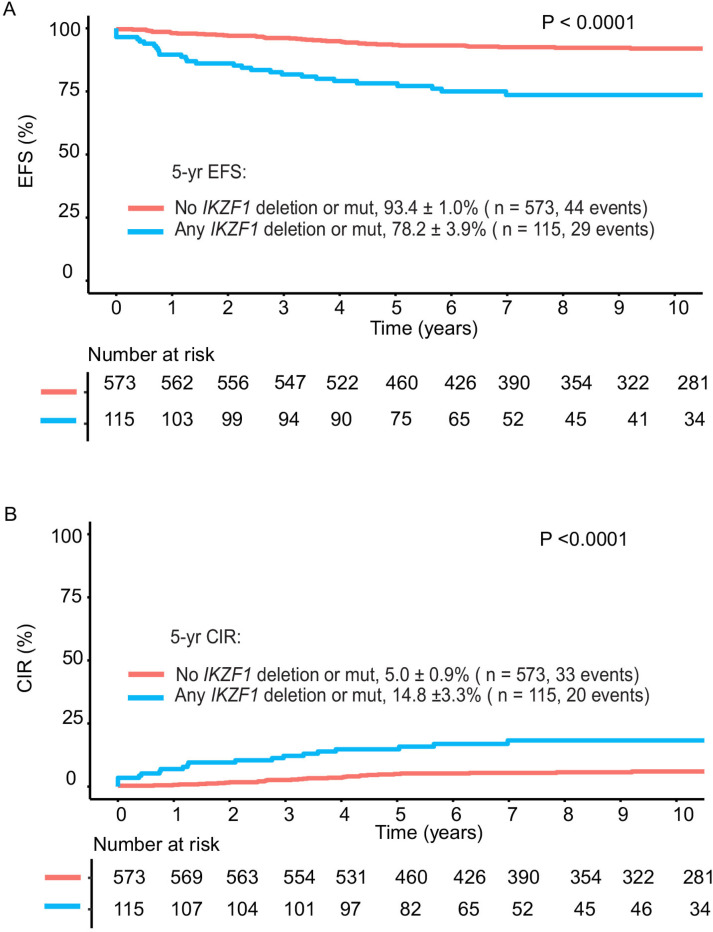
Outcomes of patients with or without any *IKZF1* alterations in the Total 15 and 16 study group. **A,** Event-free survival (EFS) and **B,** Cumulative Incidence of Relapse (CIR) for patients based on presence or absence of *IKZF1*deletion or mutations in studied patients. *IKZF1* deletion includes focal *IKZF1* deletions, −7/del(7p). mut: missense, nonsense or frameshift mutations.

**Figure 3:**
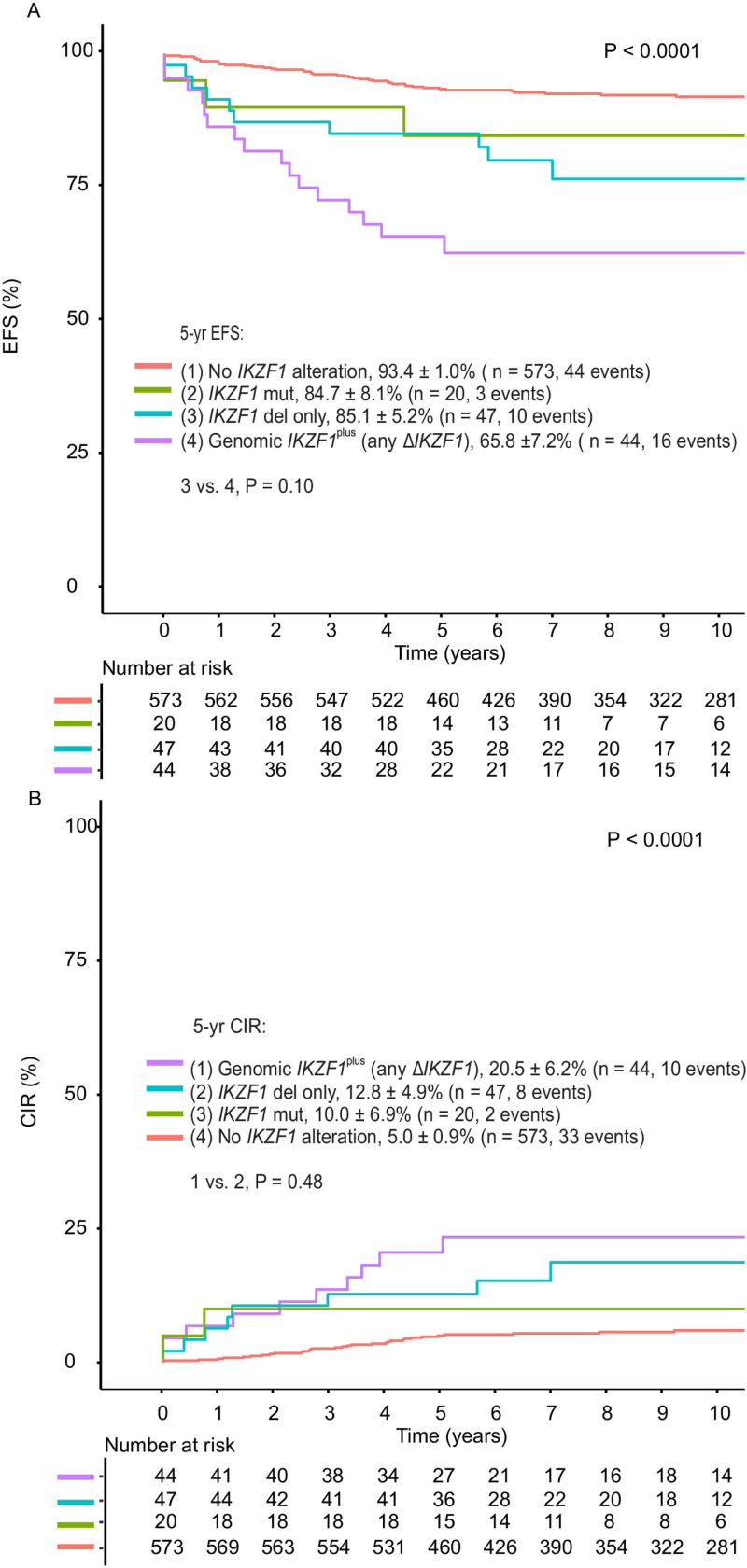
Outcomes based on type of *IKZF1* alterations, including *IKZF1*^plus^ (genomic, any Δ*IKZF1*) and sequence mutations in the Total 15 and 16 study group. **A,** Event-free survival (EFS) and **B,** Cumulative Incidence of Relapse (CIR). *P* values for pairwise comparisons for 5-year EFS compared to the no *IKZF1* alteration group are < 0.0001 for *IKZF1*^plus^ group, 0.0006 for *IKZF1* del only, and 0.19 for *IKZF1* mut. Alteration groups are mutually exclusive; data are shown for patients with only one type of alteration. *IKZF1* deletions (D*IKZF1*) are defined as focal *IKZF1* deletions or −7/del(7p). Mut: missense, nonsense or frameshift mutations.

Prior studies examining *IKZF1* alterations and outcome in ALL, including *IKZF1*^plus^ have considered − 7/del(7p) as *IKZF1* deletions. The MLPA-based definition of *IKZF1*^plus^ underestimates the frequency of *CRLF2*r and *DUX4*r as it cannot detect rearrangements directly, but uses PAR1/*ERG* deletions as surrogates for the rearrangements, which are not present in all cases.(7) To precisely define the frequency of *IKZF1* alterations among B-ALL subtypes, we examined subtypes of patients with *IKZF1* sequence mutations or no *IKZF1* alterations and patients with 1) genomic *IKZF1*^plus^ (focal Δ*IKZF1*) and focal Δ*IKZF1* only (Fig. 1A and C); 2) *IKZF1* Δ4–7 and non- *IKZF1* Δ4–7 focal deletions (Fig. 1B and C); 3) genomic *IKZF1*^plus^ (any Δ*IKZF1*) or any Δ*IKZF1* only (Fig. 1D and E); and 4) MLPA-based *IKZF1*^plus^ (any Δ*IKZF1*) (Fig. 1F).

Favorable subtypes had the lowest frequencies of *IKZF1* alterations (Fig. 1). *IKZF1* deletions were enriched in *BCR::ABL1* and *BCR::ABL1*-like (both *CRLF2*r and non-*CRLF2*r) ALL (Fig. 1). Unlike the genomic *IKZF1*^plus^ (any Δ*IKZF1*) definition in which all cases with any *CRLF2*r were identified and all cases with *DUX4*r were excluded (Fig. 1D and E), MLPA-based *IKZF1*^plus^ (any Δ*IKZF1*) misclassified 7% of *DUX4*r without *ERG* deletion as *IKZF1*^plus^ and 10% of *IKZF1*^plus^ with *CRLF2*r as *IKZF1* deletions only (Fig. 1F). Focal *IKZF1* deletions were not observed in low hypodiploid and near haploid subtypes (Fig. 1A-C). Genomic *IKZF1*^plus^ (focal Δ*IKZF1*) was identified most commonly in patients with *BCR::ABL1*-like ALL with *CRLF2*r (60%), *BCR::ABL1* ALL (30%), and *BCR::ABL1*-like ALL without *CRLF2*r (25%; Fig. 1A-C). *IKZF1* Δ4–7 deletions and *IKZF1*^plus^ overlapped in some patients, except those with *DUX4*r subtype (Fig. 1C).

### The association of IKZF1 alterations with presenting clinical features and response to induction therapy

A significantly higher frequency of genomic *IKZF1*^plus^ (any Δ*IKZF1*) was observed among patients greater than 10 years of age (*P* = 0.002) and those classified as NCI high risk (*P* = 0.0002) (Table 1). There were no significant differences in the frequency of age groups or NCI risk among patients with genomic *IKZF1*^plus^ (focal Δ*IKZF1*) (Table S7). There were no statistically significant differences in sex, race, or CNS status in patients with any type of *IKZF1* alterations compared to patients without alterations (Table 1, Table S7, and Table S8).

Genomic *IKZF1*^plus^ (any Δ*IKZF1*) (Table 1), genomic *IKZF1*^plus^ (focal Δ*IKZF1*) (Table S7), and *IKZF1* Δ4–7 (Table S8) were associated with poor response to induction therapy. A higher percentage of patients with EOI MRD ≥ 0.01% was observed in patients with *IKZF1* alterations including in 38.6% of patients with genomic *IKZF1*^plus^ (any Δ*IKZF1*), 25.5% of patients with any Δ*IKZF1* only, and 20% of patients with *IKZF1* mutations, compared to 12% of patients without *IKZF1* alterations (*P* < 0.0001; Table 1).

### The effect of IKZF1 alterations on clinical outcome and prognosis

Clinical outcomes were worse for patients with any type of *IKZF1* alteration with 5-year EFS of 78.2 ± 3.9% and 5-year CIR of 14.8 ± 3.3% compared to 93.4% ± 1% and 5.0 ± 0.9%, respectively, for patients without *IKZF1* alterations (*P* < 0.0001) (Fig. 2).

**Figure 4:**
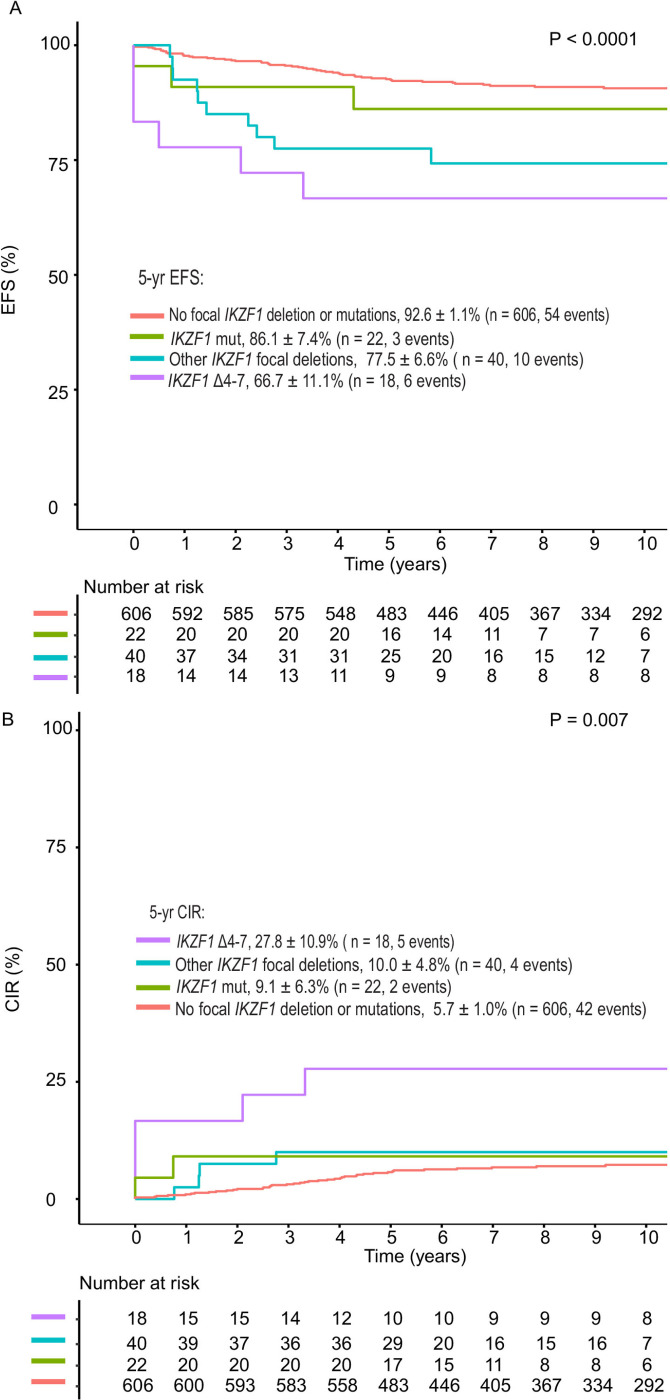
Outcomes based on presence or absence of focal *IKZF1* deletions (*IKZF1* Δ4–7 or not) or sequence mutations. **A,** Event-free survival (EFS) and **B,** Cumulative Incidence of Relapse (CIR). *P*values for pairwise comparisons for 5-year EFS compared to the no *IKZF1*alteration group are < 0.0001 for *IKZF1* Δ4–7 group, 0.0002 for other *IKZF1* focal deletions, and 0.3899 for *IKZF1* mut. Alteration groups are mutually exclusive; data are shown for patients with only one type of alteration. Mut: missense, nonsense or frameshift mutations.

Patients with genomic *IKZF1*^plus^ (any Δ*IKZF1*) and patients with any Δ*IKZF1*, without *IKZF1*^plus^, had worse outcomes than patients without *IKZF1* alterations. There were no significant differences in 5-year EFS (*P* = 0.1) and 5-year CIR (*P* = 0.48) between genomic *IKZF1*^plus^ (any Δ*IKZF1*) and any Δ*IKZF1* without *IKZF1*^plus^ (Fig. 3). Patients with genomic *IKZF1*^plus^ (focal Δ*IKZF1*) had significantly inferior 5-year EFS of 64.5 ± 8.6%, compared to 92.6 ± 1.1% (P < 0.0001) for patients without focal Δ*IKZF1* or *IKZF1* mutations. Five-year EFS did not differ significantly for patients with focal Δ*IKZF1* only (*P* = 0.07) or *IKZF1* mutations only (*P* = 0.39), compared to patients without these alterations (Table S9). *IKZF1* Δ4–7 was associated with inferior outcomes with 5-year EFS of 66.7 ± 11.1% (*P* < 0.0001) and 5-year CIR of 27.8 ± 10.9% (*P* = 0.007, Fig. 4).

We studied the effect of *IKZF1* alterations on clinical outcome and prognosis in the independent MP2PRT study group of pediatric patients with B-ALL enrolled in COG studies (Supplemental Methods and Table S2). The frequency and types of *IKZF1* alterations in the MP2PRT validation study group were similar to those in the Total 15 and 16 studies (Tables S10 and S11). There were no patients with *BCR::ABL1*, near haploid, and low hypodiploid B-ALL in the MP2PRT study group (Table S12). Additionally, there was a higher frequency of patients with *IKZF1* mutations among patients with *BCR::ABL1*-like subtypes in the MP2PRT cohort (Fig. S4). A higher percentage of patients with *IKZF1*^plus^ (any Δ*IKZF1)* or *IKZF1*^plus^ (focal Δ*IKZF1*), in the MP2PRT study group harbored WBC ≥10,000 at diagnosis and positive EOI MRD (Tables S13-S15). As observed in the Total 15 and 16 studies, clinical outcomes were worse for patients with *IKZF1* alterations in the MP2PRT study group compared to patients without *IKZF1* alterations (Fig. S5-S7). Genomic *IKZF1*^plus^ (any ΔIKZF1) was associated with 5-year EFS of 80.0 ± 5.3% compared to 94.5 ± 0.4% in patients without genomic *IKZF1*^plus^, *IKZF1* deletions or mutations (Fig. S5; P < 0.0001). *IKZF1* sequence mutations conferred significantly worse outcomes for patients in the MP2PRT study group with 5-year EFS of 87.3 ± 4.2% (Fig. S5; P = 0.02).

**Figure 5:**
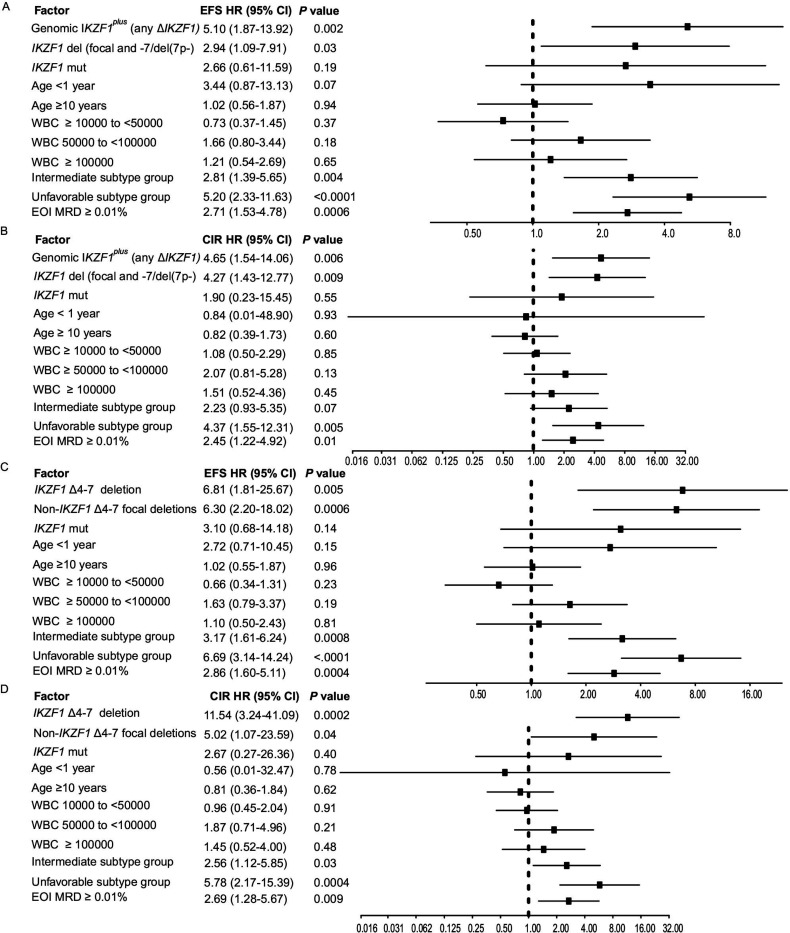
Estimated hazard ratios for event-free survival (EFS) and cumulative incidence of relapse (CIR) from Multivariable Cox Proportional Hazards Model in the Total 15 and 16 study group. **A** and **C,** Event-free survival (EFS) and **B and D**, Cumulative Incidence of Relapse (CIR) adjusting for *IKZF1* alteration status, age, genetic subtype group and EOI MRD. Comprehensive genomics-based definition of *IKZF1*^plus^ is used and *IKZF1* deletion is defined as focal *IKZF1* deletions or −7/del(7p) (A and B). All models include a time interaction term to reflect non-proportional hazards effect by the covariate *IKZF1* alteration status. *P* values for time interaction terms: A, 0.06; B, 0.11; C, 0.009; D, 0.011. WBC: presenting white blood cell count/μl, EOI MRD: end of induction minimal residual disease, Mut: nonsense, missense or frameshift mutations.

Using multivariable models, adjusting for genetic subtype group, age, presenting WBC and EOI MRD, in the Total 15 and 16 study groups, genomic *IKZF1*^plus^ (any Δ*IKZF1*) (Fig. 5A and B) or genomic *IKZF1*^plus^ (focal Δ*IKZF1*) (Table S16), *IKZF1* Δ4–7 and non-*IKZF1*Δ4–7 focal deletions (Fig. 5C and D) were independent adverse prognostic factors associated with increased hazard ratios for EFS and CIR. Unfavorable subtype group and EOI MRD ≥ 0.01% were also independent adverse prognostic factors (Fig. 5 and Table S16). As a group, *IKZF1* missense mutations were not independent prognostic factors (Fig. 5 and Table S16). *IKZF1* Δ4–7 was associated with the highest HR for CIR (11.54; 95% CI 3.24–41.09, *P* = 0.0002) for CIR (Fig. 5D). Differences in types and frequencies of B-ALL subtypes (Fig. S4 and Table S12) and related outcomes (Fig. S8) in the MP2PRT study group relative to the Total 15 and 16 studies (Fig. 1 and Fig. S3) precluded multivariable analyses incorporating outcome-based subtype groups.

**Figure 6:**
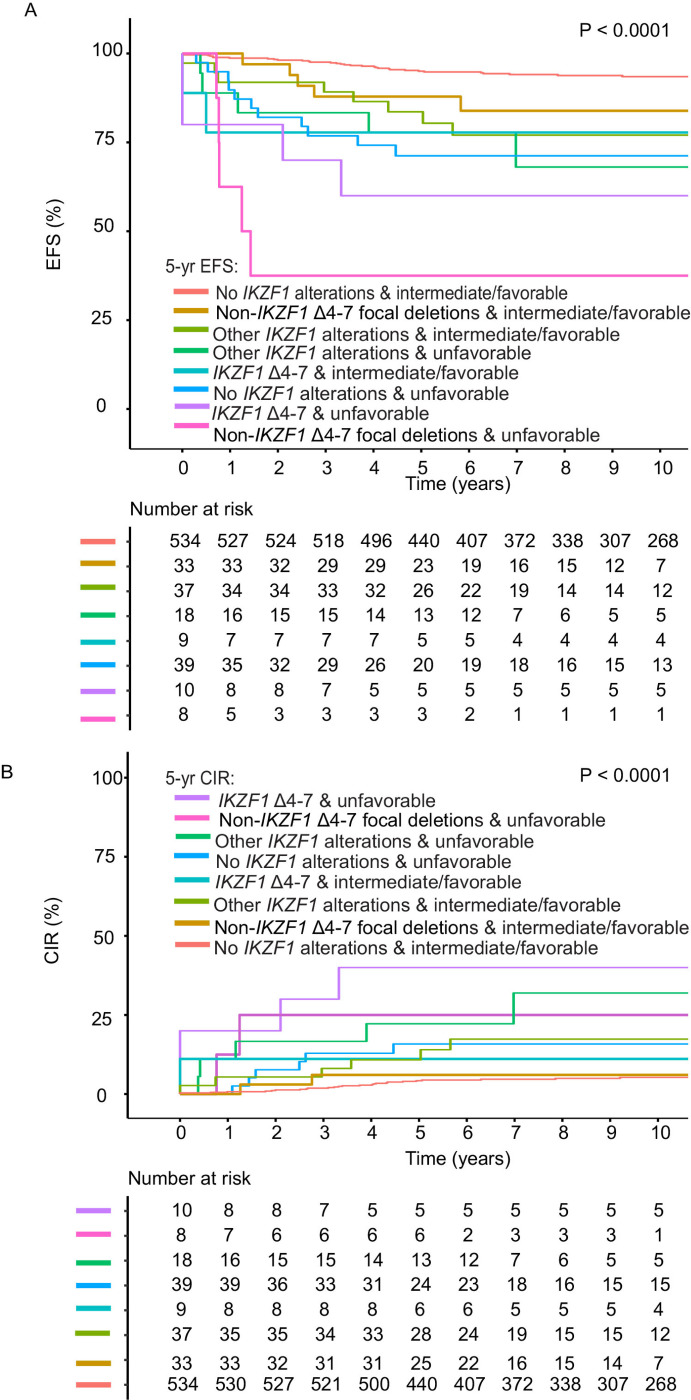
Outcomes based on type of *IKZF1* alteration (*IKZF1* Δ4–7 or not) and subtype group in the Total 15 and16 study group. **A,** Event-free survival (EFS) and **B,** Cumulative Incidence of Relapse (CIR) for patients based on type of focal *IKZF1* deletions (*IKZF1* Δ4–7 or not) or other *IKZF1* alterations (missense or frameshift mutations or −7/del(7p)), and presence or absence of unfavorable subtype group, among eligible patients with B-ALL. Five-year EFS and CIR are shown on Table S17. Alteration groups are mutually exclusive; data are shown for patients with only one type of alteration.

To determine the strongest predictors of outcome among *IKZF1* alteration groups, we examined the effect of the combination of *IKZF1* Δ4–7 and unfavorable subtype group or *IKZF1*^plus^ in the Total 15 and 16 studies. Patients with *IKZF1* Δ4–7 and unfavorable subtype group had the highest 5-year CIR (40 ± 16.5%) compared to patients without *IKZF1* Δ4–7 or unfavorable subtype group (*P* < 0.0001) (Fig. 6 Table S17). More inferior EFS observed in patients with non- *IKZF1* Δ4–7 and unfavorable subtype group is due to non-relapse events in the non- *IKZF1* Δ4–7 group (Fig. 6A). Evaluation of relapse reveals worse CIR among patients with *IKZF1* Δ4–7 and unfavorable subtype (Fig. 6B). Looking at relapse events specifically shows that patients with Ikaros D4–7 and unfavorable subtype have the worst CIR. In multivariable analyses, co-occurrence of *IKZF1* Δ4–7 and genomic *IKZF1*^plus^ was associated with HR of 11.28 for CIR (95% CI 3.32–38.35, *P* = 0.0001) (Table S18). Although limited by a small sample size, the combination of *IKZF1* Δ4–7 and unfavorable subtype group (specifically *BCR::ABL1* and *BCR::ABL1*-like with *CRLF2*r) was associated with significantly increased HR of 58.3 (95% CI 11.91–285.37, *P* < 0.0001) for CIR (Table S19). For EFS, among patients with unfavorable subtype group, the higher average HR (52.4) for patients with non- *IKZF1* Δ4–7 focal deletions vs those with *IKZF1* Δ4–7 focal deletions (40.01) was due to non-relapse events (Table S19).

*IKZF1* alterations conferred poor outcomes in patients despite undetectable EOI MRD. Patients with genomic *IKZF1*^plus^ (any Δ*IKZF1*) and undetectable EOI MRD had 5-year CIR of 15.0 ± 7.1 (*P* = 0.004) compared to patients without *IKZF1* deletions or mutations (Table S20). The 5-year CIR of 40.0 ± 25.0%; *P* < 0.0001) for patients with *IKZF1* Δ4–7 and unfavorable subtype group and negative EOI MRD was significantly worse compared to patients without *IKZF1* Δ4–7 or unfavorable subtype (Table S20).

## DISCUSSION

In this study of patients with B-ALL, we show that, in the context of risk-directed therapy and the use of tyrosine kinase inhibitors (TKI) for patients with *BCR::ABL1* leukemia, unfavorable subtype groups are independently prognostic. Our findings indicate that genomic definitions of *IKZF1*^plus^ more accurately incorporate patients with *DUX4*r and *CRLF2*r than the MLPA-based definition of *IKZF1*^plus^. Furthermore, the combination of *IKZF1* Δ4–7 and *BCR::ABL1* or *BCR::ABL1*-like subtype confers a high risk of relapse. Notably, genomic *IKZF1*^plus^ (focal Δ*IKZF1*) circumvent confounding by aneuploidy while retaining prognostic power.

Genomic *IKZF1*^plus^ (focal Δ*IKZF1*) exhibited independent adverse prognostic effect on EFS and relapse and its adverse effects on outcome were confirmed in the MP2PRT validation study group. Thus, genomic *IKZF1*^plus^ (focal Δ*IKZF1*) is a clinically informative tool for distinguishing the effects of focal *IKZF1* deletion from the effect of chromosome 7 or 7p loss. The biological differences between focal IKZF1 deletions and monosomy 7 or 7p loss are not well-understood and are likely subtype-dependent. Among B-ALL genomic subtypes, the impact of *IKZF1* haploinsufficiency has been studied in the *BCR::ABL1* subtype and, in patients, is associated with distinct gene expression (40) enrichment of Early-Pro B lineage (41) and worse outcome (41, 42). Focal *IKZF1* alterations also define distinct gene expression clusters among patients with *BCR::ABL1* subtype (30). Haploinsufficiency may result in preserved WT *IKZF1* function whereas dominant negative alleles (*IKZF1* Δ4–7 or other dominant negative point mutants) may more grossly impair *IKZF1* function (13, 43).

We highlight a significant independent adverse prognostic effect of *IKZF1* Δ4–7 on relapse and worse outcomes for patients with both *IKZF1* Δ4–7 and *BCR::ABL1* and *BCR::ABL1*-like with *CRLF2*r unfavorable subtypes, despite negative EOI MRD. Although the small sample size of patients with *IKZF1* alterations and unfavorable subtype groups is a limiting factor, our results underscore the potential utility of identifying patients with *IKZF1* Δ4–7 for intensified therapy or employing more sensitive MRD detection methods in *BCR::ABL1* and *BCR::ABL1*-like subtypes. Notably, TKI therapy was not administered to patients with *BCR::ABL1*-like subtypes in our study. Further investigation is warranted to corroborate the prognostic independence of genomically defined *IKZF1*^plus^, *IKZF1* Δ4–7 and unfavorable B-ALL subtypes in independent cohorts with larger sample sizes.

The frequencies of *IKZF1* alterations among different B-ALL subtypes may elucidate observed differences between types of *IKZF1* alterations and clinical features or outcomes. The higher frequency of patients older than 10 years within the genomic *IKZF1*^plus^ (any Δ*IKZF1*) category may, in part, stem from the inclusion of some patients with low hypodiploid B-ALL, a subtype associated with older age (44, 45), in this definition of *IKZF1*^plus^. Additionally, the higher frequency of patients with *IKZF1* mutations among patients with the unfavorable *BCR::ABL1*-nonCRLF2 subtype likely contributed to the association of *IKZF1* mutations with significantly poor 5-year EFS in the MP2PRT cohort. Notably, *IKZF1* missense mutations did not exert an independently prognostic effect on EFS or CIR upon adjusting for subtype group in the Total 15 and 16 study group. Limitations of our study include the retrospective design and small sample sizes for some genetic subtypes which preclude accurate assessment of the impact of *IKZF1* alterations within each subtype.

Although comprehensive genomic profiling is not available in many centers, detection of key abnormalities can improve the identification of *IKZF1*^plus^ cases to approximate our genomic definition. For example, to allow inclusion of all *CRLF2* and *DUX4* rearranged cases, CRLF2 and flow cytometry assays for CRLF2 overexpression and DUX4 RNA quantified. Furthermore, widely used MLPA assays can be improved with probes differentiating focal IKZF1 deletions from 7p or whole chromosome 7 losses.

In summary, our study underscores the importance of leukemia subtype classification, characterization of *IKZF1* alterations, and genomics-based analysis of cooperating lesions, in predicting the prognosis of pediatric patients with B-ALL undergoing MRD-directed therapy. Unlike genomic *IKZF1*^plus^ (any Δ*IKZF1*), genomic *IKZF1*^plus^ (focal Δ*IKZF1*) avoids overcalling *IKZF1* deletions due to aneuploidy while remaining highly predictive of relapse. *IKZF1* Δ4–7 is independently adversely prognostic, by itself or in combination with *IKZF1*^plus^ and confers the poorest outcome in patients with *BCR::ABL1* and *BCR::ABL1*-like ALL. We advocate for the inclusion of *IKZF1* Δ4–7, genomic *IKZF1*^plus^ (focal Δ*IKZF1*), and subtype grouping into risk stratification protocols to inform treatment decisions.

## Supplementary Material

Supplement 1

## Figures and Tables

**Table 1:** Clinical features of patients based on *IKZF1* alteration status, including genomic *IKZF1*^plus^ (any Δ*IKZF1*)

Clinical features		*IKZF1*^plus^ (n = 44)	*IKZF1* del only (n = 47)	*IKZF1* mut (n = 20)	No *IKZF1* alteration (n = 573)	P-value
**Age**	< 1 y	0(0.0)	0(0.0)	0(0.0)	5(0.9)	0.002*
	1 to 10 y	30(68.2)	27(57.4)	14(70.0)	468(81.7)	
	≥ 10 y	14(31.8)	20(42.6)	6(30.0)	100(17.5)	
**Race**	White	36(81.8)	39(83.0)	19(95.0)	448(78.2)	0.68*
	Black	5(11.4)	7(14.9)	1(5.0)	93(16.2)	
	Other	3(6.8)	1(2.1)	0(0.0)	32(5.6)	
**Sex**	Male	25(56.8)	26(55.3)	11(55.0)	302(52.7)	0.94
	Female	19(43.2)	21(44.7)	9(45.0)	271(47.3)	
**WBC group**	< 10000	15(34.1)	25(53.2)	12(60.0)	240(41.9)	0.05
	≥ 10000 - < 50000	17(38.6)	10(21.3)	5(25.0)	224(39.1)	
	≥ 50000 - < 100000	8(18.2)	3(6.4)	2(10.0)	64(11.2)	
	≥ 100000	4(9.1)	9(19.1)	1(5.0)	45(7.9)	
**CNS disease status**	CNS 1	31(70.5)	32(68.1)	12(60.0)	365(63.7)	0.12*
	CNS 2	10(22.7)	10(21.3)	4(20.0)	168(29.3)	
	CNS 3	2(4.5)	1(2.1)	3(15.0)	12(2.1)	
	Traumatic + blasts	1(2.3)	4(8.5)	1(5.0)	28(4.9)	
**MRD D15/19**	≥ 5%	11(26.2)	15(32.6)	7(35.0)	38(6.7)	< .0001*
	< 5%	31(73.8)	31(67.4)	13(65.0)	528(93.3)	
**EOI MRD**	≥ 0.01%	17(38.6)	12(25.5)	4(20.0)	68(12.0)	< .0001
	< 0.01%	27(61.4)	35(74.5)	16(80.0)	499(88.0)	
**HSCT**	No	36(81.8)	42(89.4)	19(95.0)	562(98.1)	< .0001*
	Yes	8(18.2)	5(10.6)	1(5.0)	11(1.9)	
**Final risk**	Low	7(15.9)	12(25.5)	6(30.0)	345(60.2)	< .0001
	Standard/High	37(84.1)	35(74.5)	14(70.0)	228(39.8)	
**B NCI RISK**	Standard	18(40.9)	18(38.3)	10(50.0)	365(63.7)	0.0002
	High	26(59.1)	29(61.7)	10(50.0)	208(36.3)	

* Alteration groups are mutually exclusive; data are shown for patients with only one type of alteration (n = 684). *P* values derived from Chi-square tests unless denoted by for Exact chi-square tests. CNS: central nervous system, HSCT: hematopoietic stem cell transplant, Mut: nonsense, missense or frameshift mutations
