# Supplementary material for: Heterogeneity of IKZF1 genomic alterations and risk of relapse in childhood B-cell precursor acute lymphoblastic leukemia
Source: Res Sq. 2024 Nov 11:rs.3.rs-5292018. Preprint. [Version 1] doi: 10.21203/rs.3.rs-5292018/v1 (PMC11601832; doi:10.21203/rs.3.rs-5292018/v1)
Supplement: Supplement 1 [file NIHPPRS5292018V1-supplement-1.pdf]

Supplementary Files

This is a list of supplementary files associated with this preprint. Click to download.

- DataSupplementCleanFinal.docx
- SupplementalDataTableS1.pdf
- SupplementalDataTableS2.pdf
- SupplementalDataTableS3.pdf
- SupplementalDataTableS4.pdf
- SupplementalDataTableS5.pdf
- SupplementalDataTableS6.pdf
- SupplementalDataTableS7.pdf
- SupplementalDataTableS8.pdf
- SupplementalDataTableS9.pdf
- SupplementalDataTableS10.pdf
- SupplementalDataTableS11.pdf
- SupplementalDataTableS12.pdf
- SupplementalDataTableS13.pdf
- SupplementalDataTableS14.pdf
- SupplementalDataTableS15.pdf
- SupplementalDataTableS16.pdf
- SupplementalDataTableS17.pdf
- SupplementalDataTableS18.pdf
- SupplementalDataTableS19.pdf
- SupplementalDataTableS20.pdf
